# Feasibility of using Clinical Practice Research Datalink data to identify patients with chronic obstructive pulmonary disease to enrol into real‐world trials

**DOI:** 10.1002/pds.5188

**Published:** 2021-01-09

**Authors:** Gema Requena, Achim Wolf, Rachael Williams, Daniel Dedman, Jennifer K. Quint, Tarita Murray‐Thomas, Jeanne M. Pimenta

**Affiliations:** ^1^ Respiratory Epidemiology GlaxoSmithKline Brentford UK; ^2^ Clinical Practice Research Datalink Medicines and Healthcare Products Regulatory Agency London UK; ^3^ Respiratory Epidemiology, Occupational Medicine and Public Health National Heart and Lung Institute, Imperial College London London UK

**Keywords:** chronic obstructive pulmonary disease, electronic health record, feasibility studies, general practice, primary health care, real world clinical trials

## Abstract

**Purpose:**

To assess the feasibility of using Clinical Practice Research Datalink (CPRD) data for identifying populations of patients with chronic obstructive pulmonary disease (COPD) eligible for a hypothetical pragmatic trial.

**Methods:**

A retrospective multidatabase cohort study using CPRD primary care and linked secondary care data to describe the characteristics of populations of patients with COPD. Patients' demographic and lifestyle factors, comorbidity profile, spirometry measurements and treatment changes were evaluated, as was the distribution of follow‐up time and types of losses during follow‐up. Characteristics were evaluated using descriptive statistics.

**Results:**

A total of 322 991 patients from 1148 primary care practices in the United Kingdom across two CPRD primary care databases, CPRD GOLD and CPRD Aurum, were potentially eligible to participate in a hypothetical trial using CPRD, starting on 31 December 2017. Patients with COPD in CPRD GOLD and CPRD Aurum were comparable in terms of age (median age 70 vs. 68 years), gender (50% vs. 52% male), disease severity (e.g., 25% vs. 24% Medical Research Council [MRC] dyspnoea score grades 3–5) and history of respiratory conditions (e.g., 43% vs. 38% asthma). High proportions of patients with COPD in CPRD GOLD and CPRD Aurum were available on 31 December 2012 for follow‐up at 1, 2, and 5 years (92%, 85% and 67%, respectively).

**Conclusions:**

Patients and data from CPRD GOLD and CPRD Aurum were comparable across key aspects relevant to COPD trials. A pragmatic trial using CPRD to recruit patients with COPD is scientifically feasible.

**Key Points**

- Real‐world data from primary‐care electronic health records allows for identification of a large, well‐characterised cohort of patients often used to undertake safety studies, long‐term natural history studies or comparative effectiveness research.
- Data from the Clinical Practice Research Datalink (CPRD) GOLD and CPRD Aurum databases were analysed to evaluate the feasibility of identifying patients with chronic obstructive pulmonary disease (COPD) for enrolment into real‐world trials.
- A total of 322 991 patients from 1148 general practices in the United Kingdom were identified from CPRD databases for potential trial enrolment using a case study approach.
- Patients and data from the CPRD GOLD and CPRD Aurum primary care databases were broadly comparable across key aspects relevant to a COPD trial.
- A pragmatic trial using the CPRD to recruit patients with COPD is scientifically feasible.

## 1 INTRODUCTION

Pragmatic trials test the real‐world effectiveness of treatments with the aim of informing clinical practice, allowing diverse populations to be studied and providing good external generalisability of the trial results.^1^ Interest in these trials has increased, due partially to technology advances and increased use of electronic health records (EHRs).^2^, ^3^ The Salford Lung Study (SLS) is an example of a real‐world pragmatic trial with broad inclusion/limited exclusion criteria, providing data with direct clinical applicability. The Salford Integrated Record was used to link primary and secondary care data in the SLS, providing comprehensive patient‐level EHRs.^4^

The Clinical Practice Research Datalink (CPRD) is a real‐world research service supporting retrospective clinical studies.^5^ CPRD collect, clean and process de‐identified patient data using EHRs from a sample of general practitioner (GP), that is, primary care, practices in the UK that use either the Vision or EMIS software systems contributing to the CPRD GOLD or CPRD Aurum primary care databases, respectively.^6^, ^7^ These de‐identified databases have been individually linked to secondary care and other health‐ and area‐based datasets.^5^ EHR data can be linked to a trial database for interventional trials.^8^, ^9^

CPRD GOLD has been used previously for observational respiratory research and validated algorithms are available to identify patients with chronic obstructive pulmonary disease (COPD).^10^ It has also been used to determine outcomes such as acute exacerbations of COPD with high specificity and positive predictive value.^10^, ^11^ However, observational validation studies have yet to be replicated using CPRD Aurum and to date no pragmatic trials in patients with COPD have been conducted using either CPRD database. The overall aim of the current study was to assess the feasibility of using CPRD data to enrol patients in a hypothetical future trial comparing the real‐world effectiveness and safety of newly authorised COPD maintenance therapies.

### 1.1 Objectives

The study had three specific objectives: (1) to estimate the number of primary care patients with COPD in CPRD databases, for whom data were being actively collected from GP practices on 31 December 2017, to inform the number of patients eligible to participate in a hypothetical trial; (2) to describe the characteristics of patients with COPD and their GP practices in the 12 months before potential enrolment on 31 December 2017; (3) to describe the follow‐up time, reasons for loss to follow‐up and mortality rates up to 5 years, in a sub‐cohort of patients with COPD who were actively registered in CPRD practices on 31 December 2012, and whose GP practices still contributed to CPRD on 31 December 2017, to understand how long patients might remain in a study after enrolment.

## 2 METHODS

### 2.1 Study design

In this retrospective study, patients with COPD were identified based on diagnosis codes recorded in primary care using an algorithm previously validated in CPRD GOLD.^10^ A cross‐sectional design was used to enumerate and describe the population registered in CPRD on 31 December 2017 (objectives 1 and 2). A cohort design was used to describe the distribution of follow‐up time and the types of loss‐to‐follow‐up in patients who would have been eligible to participate in the hypothetical pragmatic trial on 31 December 2012 (objective 3).

### 2.2 Data sources

UK primary care data from CPRD GOLD (December 2018 release) and English primary care data from CPRD Aurum (January 2019 release) were analysed. CPRD GOLD included 16 million individuals (with acceptable quality medical records) from 1987 onwards, from whom data were actively being collected for 2.2 million patients. CPRD Aurum included 22 million individuals, from whom data were being actively collected for 7.3 million patients. Coded diagnostic data from the CPRD person‐level deterministically‐linked Hospital Episode Statistics (HES) Admitted Patient Care data were also analysed. National Health Service (NHS) Digital performed linkage of CPRD data to HES using an 8‐stage deterministic methodology. Study investigators had full access to the databases used to create the study population.

### 2.3 Study populations

**FIGURE 1:**
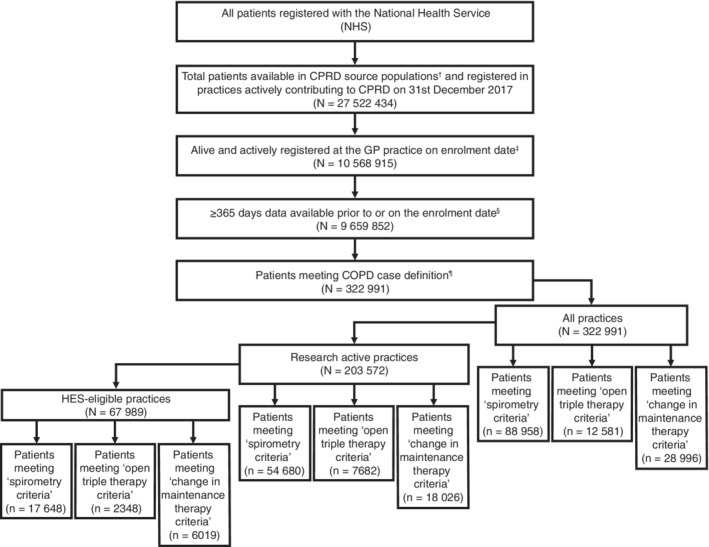
Flowchart of patients in the study. COPD, chronic obstructive pulmonary disease; CPRD, Clinical Practice Research Datalink; FEV_1_, forced expiratory volume in 1 s, FVC, forced vital capacity; GP, general practitioner; HES, hospital episode statistics; ICS, inhaled corticosteroid; LABA, long‐acting beta‐2 agonist; LAMA, long‐acting muscarinic antagonist. ^†^Six source populations were: (1) practices contributing to CPRD GOLD, (2) practices contributing to CPRD Aurum, (3) research‐active practices contributing to CPRD GOLD, (4) research‐active practices contributing to CPRD Aurum, (5) research‐active practices contributing to CPRD GOLD that are also eligible for linkage with secondary‐care data (HES) and (6) research‐active practices contributing to CPRD Aurum that are also eligible for linkage with secondary‐care data (HES). ^‡^Enrolment dates: For objectives 1 and 2 (enumeration and description of the populations), the enrolment date is 31 December 2017; for objective 3 (description of distribution of follow‐up time and the types of loss‐to‐follow‐up), it is 31 December 2012. ^§^For CPRD GOLD, the requirement was ≥365 days of up‐to‐standard data available. ^¶^Patients met the COPD case definition if they had a COPD clinical code defined by Quint et al^10^ in all available history prior to or on the enrolment date and were ≥ 35 years old on enrolment date. Note: The criteria for spirometry and maintenance therapy are not mutually exclusive. *Spirometry subgroup*: Patients were required to have a ratio of FEV_1_/FVC measurements of <0.7 recorded at any time on or prior to enrolment date. *Treatment change subgroup*: Patients were required to have received ≥1 prescription for long‐acting COPD maintenance inhalation therapy (e.g., LABA with or without ICS) in the 12 months on or prior to enrolment with evidence of treatment change (initiation of a specific active substance or combination of active substances) in the 6 months on or prior to the hypothetical enrolment date of 31 December 2017. *Open triple therapy subgroup*: Patients were required to have been treated continuously with open triple therapy (ICS, LABA and LAMA; excluding fluticasone furoate/vilanterol trifenatate/umeclidinium bromide) for a duration of at least 3 months on or prior to hypothetical enrolment with a documented history of at least one moderate or severe exacerbation (as defined in Table S1 in Data S1) in the year on or prior to 31 December 2017

The study population comprised patients with COPD registered in CPRD GOLD and CPRD Aurum practices in the UK, including patients in research active practices and in those eligible for linkage to HES data, forming six total source populations (Figure 1). Patients met the study eligibility criteria if they had a COPD clinical code^10^ in all available history prior to, or on, the enrolment date and were ≥35 years old on enrolment date. Practices were counted as “research active” if they had Royal College of GPs Research Ready accreditation, including clinical trials capability and/or previous participation in biosample, patient‐reported outcomes, cluster randomised clinical trials, pragmatic clinical trials, or clinical trial participant identification studies with CPRD. Only patients from research‐active practices would be eligible for enrolment in potential future real‐world trials. Patients in research‐active practices eligible for linkage to HES data were from practices in England only. Analyses were repeated in three COPD subgroups of interest: patients meeting spirometry criteria, change in maintenance therapy criteria and open triple therapy (“open” defined as therapy delivered via multiple inhalers, rather than a single inhaler) criteria (Figure 1). The subgroups were aligned with case definitions that may be applied in future pragmatic clinical trials. Objectives 1 and 2 only were evaluated in the open, triple therapy subgroup, as this therapy was a relatively new treatment regimen at the time of study.

### 2.4 Variables

Practice characteristics, patient demographics, respiratory history, disease severity, healthcare utilisation and respiratory medications were measured in general practices contributing to CPRD GOLD and CPRD Aurum and in patients who met the study eligibility criteria. Read code lists to identify the covariates and outcomes were based on three previous studies of CPRD data.^10^, ^11^, ^12^ Medical conditions were identified using clinical codes from CPRD GOLD and CPRD Aurum. Details on data sources and method of assessment for each variable can be found in Table S1 in Data S1.

### 2.5 Ethics

GP practices provided consent for CPRD to collect their patients' de‐identified data. Individual patients could opt‐out of contributing data to CPRD. GP practices provided consent for data to be linked to HES. No patient identifiable information was available to the study team, or to the study sponsor, GlaxoSmithKline plc. This study was approved by the Independent Scientific Advisory Committee for MHRA database research, protocol number 17_066A.

### 2.6 Analyses

This study was descriptive and no statistical hypotheses were tested. Analyses were conducted separately for CPRD GOLD and CPRD Aurum practices to describe the number of patients in each database and determine whether the demographic characteristics of patients differed between CPRD GOLD and Aurum, and whether information was recorded at similar levels in the two software systems. Analyses were conducted for COPD subpopulations in each of the six source populations (Figure 1). Characteristics were measured as number (percentage) for non‐continuous variables, mean (standard deviation) and median (interquartile range) for continuous variables. The lung function/airflow limitation was summarised by the forced expiratory volume in 1 s (FEV_1_) percent predicted, using the Global Initiative for Chronic Obstructive Lung Disease (GOLD) 2006 definitions^13^ [GOLD 1: FEV_1_% predicted ≥80%, GOLD 2: FEV_1_% predicted ≥50%−<80%, GOLD 3: FEV_1_% predicted ≥30%−<50%, GOLD 4: FEV_1_% predicted <30%], measured using nearest record prior to or on the enrolment date.

The sub‐cohort of patients with COPD actively registered in the practices on 31 December 2012 were described and followed up until death, leaving the GP practice or 31 December 2017, whichever was earliest, to understand how long patients may remain in a potential pragmatic trial. The mean, median, interquartile range of follow‐up time and mortality rate (per 1000 person years) were described. The number and proportion of patients who remained actively registered at their GP practice at 1, 2, 3, 4 and 5 years after 31 December 2012 were also described. Start of patient follow up was defined as the latest of current registration date and practice up‐to‐standard date. End of patient follow‐up was defined as the earliest of patient transfer out date, CPRD GOLD‐derived death date or CPRD Aurum death date and practice last collection date.

An “unknown” category was created for variables with missing data. Missing data for lung function/airflow limitation were imputed using a combination of FEV_1_ (litres), height, gender and age.

The data in the study are reported, and current manuscript developed, in line with the REporting of studies Conducted using Observational Routinely‐collected health Data (RECORD) statement.^14^

## 3 RESULTS

### 3.1 Objective 1: Patient numbers

Across both the CPRD GOLD and CPRD Aurum source populations, a total of 322 991 patients from 1148 practices were included in the analyses (Table 1). The CPRD Aurum population was considerably larger than the CPRD GOLD population, comprising 82% of the total source population and 74% of all source practices.

**TABLE 1:** Number of practices and patients with COPD registered in CPRD‐GOLD and CPRD Aurum on 31 December 2017, stratified by practice type and subpopulation

	CPRD GOLD			CPRD Aurum			Total (all practices)
	All practices	Research active	HES‐eligible	All practices	Research active	HES‐eligible	
Practices, N	295	144	46	853	522	169	1148
Patients, N	56 813	29 027	9804	266 178	174 545	58 185	322 991
Spirometry subgroup, n (%)	21 086 (37)	11 219 (39)	3976 (41)	67 872 (26)	43 461 (25)	13 672 (24)	88 958 (28)
Treatment change subgroup, n (%)	6323 (11)	3227 (11)	1086 (11)	22 673 (9)	14 799 (9)	4933 (9)	28 996 (9)
Open triple therapy subgroup, n (%)	3600 (6)	1951 (7)	725 (7)	8981 (3)	5731 (3)	1623 (3)	12 581 (4)

*Note:* Spirometry subgroup: Patients were required to have a ratio of forced expiratory volume in 1 s (FEV_1_)/forced vital capacity (FVC) measurements of <0.7 recorded at any time on or prior to enrolment date. Treatment change subgroup: Patients were required to have received ≥1 prescription for long‐acting COPD maintenance inhalation therapy in the 12 months on or prior to enrolment with evidence of treatment change (initiation of a specific active substance or combination of active substances) in the 6 months on or prior to the hypothetical enrolment date of 31 December 2017. Open triple therapy subgroup: Patients were required to have been treated continuously with open triple therapy (excluding fluticasone furoate/vilanterol trifenatate/umeclidinium bromide) for a duration of at least 3 months on or prior to hypothetical enrolment with a documented history of at least one moderate or severe exacerbation (as defined in Table S1 in Data S1) in the year on or prior to 31 December 2017.

Abbreviations: COPD, chronic obstructive pulmonary disease; CPRD, Clinical Practice Research Datalink; HES, hospital episode statistics.

### 3.2 Objective 2: Patient characteristics

#### 3.2.1 CPRD GOLD versus CPRD Aurum (in all practices)

In all practices, patients with COPD in CPRD GOLD and CPRD Aurum were numerically comparable in terms of age (70 vs. 68 years median age), gender (50% vs. 52% male), and deprivation (50% vs. 49% in most deprived two quintiles; England only). Patients were also comparable in terms of smoking status (38% vs. 40% current smokers) and body mass index (BMI) (60% vs. 59% overweight or obese), though fewer patients in CPRD GOLD had no recorded BMI (7% vs. 19% in CPRD Aurum).

Proportions of patients with a history of respiratory conditions were also numerically comparable in the CPRD GOLD and CPRD Aurum all‐practice source populations, including for asthma (43% vs. 38%), bronchiectasis (5% vs. 5%), and pneumonia (8% vs. 10%). Measures of disease severity, including Medical Research Council (MRC) grades 3–5 (25% vs. 24%); Lung function, FEV_1_% predicted, GOLD grades 3–4, (17% vs. 16%); median FEV_1_/FVC ratio (63 vs. 67); and moderate exacerbation episodes (28% vs. 22%) were also similar between these populations. Patients in both data sources had comparable mean numbers of GP visits but patients in CPRD GOLD had higher numbers of practice nurse visits and flu vaccination rates (Table 2).

**TABLE 2:** Selected characteristics in all practices, research‐active practices and HES‐eligible practices of patients with COPD registered in CPRD GOLD and CPRD Aurum on 31 December 2017

Characteristic	All practices		Research active		HES‐eligible	
Total patients, *N*	322 991		203 572		67 989	
Database (Patients, *N*)	CPRD GOLD (56 813)	CPRD Aurum (266 178)	CPRD GOLD (29 027)	CPRD Aurum (174 545)	CPRD GOLD (9804)	CPRD Aurum (58185)
Male	28 673 (50)	137 743 (52)	14 640 (50)	89 864 (52)	4708 (48)	30 172 (52)
Age in years, median (IQR)	70 (62–77)	68 (57–76)	70 (62–78)	68 (57–77)	71 (63–78)	68 (57–77)
Deprivation (top two quintiles)^a^	10 934 (50)	129 573 (49)	5993 (48)	75 895 (44)	4574 (47)	21 363 (37)
Current smokers^b^	21 362 (38)	105 953 (40)	10 411 (36)	69 164 (40)	3296 (34)	21 873 (38)
BMI^b^ Underweight (<18.5)	2429 (4)	6210 (2)	1233 (4)	4018 (2)	413 (4)	1315 (2)
Normal (≥18.5 and <25)	16 224 (29)	53 999 (20)	8327 (29)	36 020 (21)	2932 (30)	12 197 (21)
Overweight (≥25 and <30)	17 254 (30)	62 338 (24)	8804 (30)	41 608 (24)	2976 (30)	14 069 (24)
Obese (≥30 and <70)	16 755 (30)	94 064 (35)	8458 (29)	60 341 (34)	2940 (30)	19 655 (34)
History of asthma	24 409 (43)	102 459 (38)	12 484 (43)	66 592 (38)	4567 (47)	22 529 (39)
History of bronchiectasis	2958 (5)	12 192 (5)	1526 (5)	7849 (5)	546 (6)	2615 (5)
History of pneumonia	4583 (8)	27 501 (10)	2498 (9)	18 280 (11)	771 (8)	6188 (11)
MRC grades 3–5^b^	14 356 (25)	64 325 (24)	7365 (25)	41 139 (24)	2797 (29)	13 784 (24)
GOLD grades 3–4^b^	9781 (17)	41 278 (16)	5208 (18)	27 060 (16)	2164 (22)	8945 (15)
FEV_1_/FVC ratio, median (IQR)	63 (53–71)	67 (56–77)	62 (52–70)	67 (56–77)	62 (52–71)	68 (57–78)
Initiation of LAMA^c^	2833 (5.0)	8692 (3.3)	1457 (5.0)	5645 (3.2)	507 (5.2)	1789 (3.1)
Initiation of LABA^c^	255 (0.45)	1203 (0.45)	146 (0.50)	823 (0.47)	53 (0.54)	312 (0.54)
Initiation of LAMA/LABA combination^c^	1580 (2.8)	4320 (1.6)	746 (2.6)	2840 (1.6)	197 (2.0)	876 (1.5)
Initiation of ICS/LABA fixed combination^c^	1897 (3.3)	7128 (2.7)	998 (3.4)	4563 (2.6)	340 (3.5)	1583 (2.7)
Initiation of ICS or ICS/SABA^c^	653 (1.2)	4264 (1.6)	327 (1.1)	2842 (1.6)	119 (1.2)	991 (1.7)
Initiation of theophylline^c^	144 (0.25)	406 (0.15)	59 (0.20)	249 (0.14)	23 (0.23)	74 (0.13)
Initiation of OCS^c^	6214 (10.9)	21 282 (8.0)	3242 (11.2)	13 716 (7.9)	1025 (10.5)	4402 (7.6)
≥1 COPD exacerbations						
Moderate	15 994 (28)	57 377 (22)	8688 (30)	37 086 (21)	3104 (32)	11 976 (21)
Severe	‐	‐	‐	‐	791 (8.1)	3097 (5.3)
Myocardial infarction	4709 (8.3)	16 775 (6.3)	2328 (8.0)	10 874 (6.2)	745 (7.6)	3518 (6.1)
Stroke	6077 (10.7)	25 276 (9.5)	3148 (10.9)	17 125 (9.8)	1028 (10.5)	5352 (9.2)
Coronary artery bypass grafts	1482 (2.6)	6296 (2.4)	743 (2.6)	4087 (2.3)	273 (2.8)	1362 (2.3)
GP visits, mean (SD)	7.6 (8.0)	6.5 (7.0)	7.1 (7.3)	6.4 (6.8)	6.4 (6.5)	6.5 (7.1)
Practice nurse visits, mean (SD)	3.6 (5.5)	1.5 (3.7)	3.5 (5.2)	1.5 (3.6)	3.3 (5.2)	1.5 (3.9)
Flu vaccination rate	41 648 (73)	176 036 (66)	21 643 (75)	115 924 (66)	7397 (75)	37 993 (65)

*Note:* Data reported are *n* (%), unless otherwise stated.

Abbreviations: BMI, body mass index; COPD, chronic obstructive pulmonary disease; CPRD, Clinical Practice Research Datalink; HES, hospital episode statistics; FEV_1_, forced expiratory volume in 1 second; FVC, forced vital capacity; GOLD, Global Initiative for Chronic Obstructive Lung Disease; GP, general practitioner; ICS, inhaled corticosteroids; IQR, interquartile range; LABA, long‐acting beta‐2 agonists; LAMA, long‐acting muscarinic antagonists; MRC, Medical Research Council; OCS, oral corticosteroids; SABA, short‐acting beta‐2 agonists; SD, standard deviation.

^a^ Deprivation is reported for England only; English practices comprised 39% of CPRD GOLD and 100% of CPRD Aurum.

^b^ Missing data for all practices, n (%). Current smokers: 212 (0.37) for CPRD GOLD and 1720 (0.65) for CPRD Aurum. BMI: 4151 (7) for CPRD GOLD and 49 567 (19) for CPRD Aurum. Dyspnoea (MRC grades): 24 902 (44) for CPRD GOLD and 110 905 (42) for CPRD Aurum. Lung function/airflow limitation (GOLD grades): 21 912 (39) and 140 646 (53) for CPRD Aurum.

^c^ Class initiated in the 6 months prior to or on the enrolment date.

Compared with CPRD Aurum, patients in CPRD GOLD had numerically higher proportions of initiation (class initiated in the 6 months prior to or on the enrolment date) of long‐acting muscarinic antagonist (LAMA)/long‐acting beta‐2 agonist (LABA) combination, inhaled corticosteroid (ICS)/LABA fixed combination, theophylline, and oral corticosteroid, but not LABA. However, patients in CPRD GOLD had numerically lower proportions of initiation of ICS or ICS/short‐acting beta‐2 agonist (SABA) (Table 2).

### 3.3 CPRD GOLD versus CPRD Aurum by type of practices

Patients in CPRD GOLD and CPRD Aurum research‐active practices and a subset of additionally HES‐eligible practices were comparable with those in all practices, across all domains measured, including the three measures of disease severity; spirometry, treatment change and open triple therapy (Table 2). Compared with CPRD Aurum, CPRD GOLD had lower proportions of research active practices (52% vs. 66%) and additionally HES‐eligible practices (17% vs. 22%).

Patients with one or more severe COPD exacerbations recorded in HES were rare for patients in CPRD GOLD and CPRD Aurum compared with moderate COPD exacerbations recorded in primary care (Table 2). Myocardial infarction, stroke and coronary artery bypass grafts were recalculated using events recorded in either primary care or HES, but results were numerically similar to those recorded solely in primary care (CPRD GOLD: myocardial infarction: 8.6% vs. 7.6%, stroke: 10.9% vs. 10.5%, coronary artery bypass grafts: 3.2% vs. 2.8 in primary care or HES vs. primary care only; CPRD Aurum: myocardial infarction: 6.8% vs. 6.1%, stroke: 9.4% vs. 9.2%, coronary artery bypass grafts: 2.6% vs. 2.3%). The mean number of all‐cause hospitalisations recorded in HES were slightly higher in CPRD GOLD than primary care (1.0 vs. 0.7). Comparatively, the mean was slightly lower in CPRD Aurum than primary care (0.7 vs. 1.0).

### 3.4 CPRD GOLD versus CPRD Aurum by subgroups

In both databases, patients in the spirometry, treatment change, and open triple therapy subpopulations were comparable with the all‐patients group in terms of age, gender, and deprivation (Table 3). Patients in the spirometry group were generally comparable in terms of comorbidities at baseline and healthcare utilisation, but rates of these variables were elevated in the treatment change and triple therapy groups (Table 3).

**TABLE 3:** Selected characteristics in all practices of patients with COPD registered in CPRD GOLD and CPRD Aurum on 31 December 2017, stratified by subpopulation

	CPRD GOLD				CPRD Aurum			
Population	All patients	Spirometry	Treatment change	Open triple therapy	All patients	Spirometry	Treatment change	Open triple therapy
Patients, *N*	56 813	21 086	6323	3600	266 178	67 872	22 673	8981
Male, *n* (%)	28 673 (50)	9862 (47)	3163 (50)	1991 (55)	137 743 (52)	29 282 (43)	11 387 (50)	4861 (54)
Age in years, median (IQR)	70 (62–77)	70 (63–77)	69 (60–76)	70 (63–77)	68 (57–76)	71 (63–77)	68 (59–76)	71 (63–78)
Deprivation (top two quintiles), n (%)^a^	10 934 (50)	4362 (48)	1173 (49)	765 (52)	129 573 (49)	34 546 (51)	11 342 (50)	5247 (58)
Myocardial infarction, *n* (%)	4709 (8.3)	1627 (7.7)	543 (8.6)	293 (8.1)	16 775 (6.3)	5224 (7.7)	1648 (7.3)	763 (8.5)
Stroke, n (%)	6077 (10.7)	2010 (9.5)	703 (11.1)	395 (11.0)	25 276 (9.5)	6761 (10.0)	2318 (10.2)	1085 (12.1)
Coronary artery bypass grafts, n (%)	1482 (2.6)	507 (2.4)	164 (2.6)	76 (2.1)	6296 (2.4)	1874 (2.8)	621 (2.7)	230 (2.6)
GP visits, mean (SD)	7.6 (8.0)	7.3 (7.3)	8.4 (8.0)	10.3 (9.3)	6.5 (7.0)	7.5 (7.1)	8.3 (7.5)	10.4 (8.8)
Practice nurse visits, mean (SD)	3.6 (5.5)	4.0 (5.3)	4.3 (5.7)	4.5 (5.4)	1.5(3.7)	2.0 (4.0)	2.2 (4.3)	2.4 (4.7)
Flu vaccination rate, n (%)	41 648 (73)	16 568 (79)	4783 (76)	2920 (81)	176 036 (66)	55 320 (82)	17 605 (78)	7544 (84)

Abbreviations: CPRD, Clinical Practice Research Datalink; GP, general practitioner; IQR, interquartile range; SD, standard deviation.

^a^ Deprivation is reported for England only; English practices comprised 39% of CPRD GOLD and 100% of CPRD Aurum.

### 3.5 Objective 3: Follow‐up time, reasons for loss‐to‐follow‐up and mortality rates

Across both source populations, 281 044 patients from 1133 practices formed the cohort of patients with COPD diagnosed prior to 31 December 2012 and who were in practices still contributing to CPRD on 31 December 2017. In both CPRD GOLD and CPRD Aurum, two‐thirds of patients with COPD were followed up for the full 5 years, with a mean follow‐up of 4 years. Three‐quarters of patients had a follow‐up of 3 years or more (Table 4). Death was a more common reason for loss‐to‐follow‐up (19% in CPRD Aurum, 27% in CPRD GOLD) than transfer out of the practice (14% in CPRD Aurum, 11% in CPRD GOLD) (Table S2 in Data S1). Similar follow up was observed in the patients in the spirometry subpopulation, treatment change subpopulation, research active subgroups, and HES subgroups.

**TABLE 4:** Number of patients with COPD by practice type in a sub‐cohort of patients with COPD who were actively registered in CPRD practices on 31 December 2012 and number and of patients who remained actively registered at their GP practice at 1, 2, 3, 4 and 5 years after 31 December 2012

Practice, *N* Patients (*N* Practices)	CPRD GOLD	CPRD Aurum	Total	Percentage of total enrolled on 31 December 2017
All practices	45 940 (280)	235 104 (853)	281 044 (1133)	87 (99)
Research‐active	23 147 (137)	152 267 (522)	175 414 (659)	86 (99)
HES‐eligible	8190 (44)	50 918 (169)	59 108 (213)	87 (99)
**Active follow‐up at (all practices), *n* patients (%):**				
**1 year**	41 858 (91)	215 724 (92)	257 582 (92)	
**2 years**	38 290 (83)	199 602 (85)	237 892 (85)	
**3 years**	34 795 (76)	184 871 (79)	219 666 (78)	
**4 years**	31 620 (69)	171 263 (73)	202 883 (72)	
**5 years**	28 723 (63)	158 735 (68)	187 458 (67)	

Abbreviations: COPD, chronic obstructive pulmonary disease; CPRD, Clinical Practice Research Datalink; GP, general practitioner; HES, hospital episode statistics.

## 4 DISCUSSION

This study assessed the feasibility of using CPRD data to identify patients with COPD to enrol into potential future trials. Our results indicated that a substantial number of patients with COPD (322 991 across CPRD GOLD and CPRD Aurum) were potentially eligible for inclusion in a hypothetical pragmatic trial, using CPRD data. Around two‐thirds of these patients (203 572) were from research‐active practices, with approximately one‐third of these patients (67 989) having records additionally eligible for HES linkage. CPRD Aurum significantly increased the patient pool; over 80% of patients were from CPRD Aurum practices, especially among research‐active practices, and offered higher geographical representativeness within England. Patients and data from CPRD GOLD and CPRD Aurum were broadly comparable across key aspects relevant to a COPD trial. Our study builds upon a small study by Quint et al,^15^ which established the feasibility of using the CPRD to screen, locate and recruit pre‐screened participants with COPD for research.

A higher proportion of patients in CPRD GOLD met the open triple therapy criteria (6%) compared with CPRD Aurum patients (3%). This is consistent with the higher proportions of therapy initiation in CPRD GOLD for all medication groups except LABA and ICS or ICS/SABA. The rates of therapy initiation in CPRD GOLD are generally reflected in the prescribing rates for COPD medications in the UK, where increases in LAMA, LAMA/LABA and triple therapy (ICS/LAMA/LABA) have been observed from 2000–2016.^16^ However, it is unclear if the differences seen in CPRD Aurum are a reflection of prescribing differences (e.g., higher use of ICS/SABA), or different recording practices caused by the GP information technology software systems (e.g., differences in recording issue dates of repeat prescriptions).

The cohort study of patients with COPD in CPRD GOLD and CPRD Aurum on 31 December 2012 found that large numbers of patients were available for follow‐up at 1, 2 and 5 years. Long‐term follow up of patients allows for study of longer‐term outcomes, including effectiveness and safety over a longer period of time and identification of rarer safety signals.^17^ For example, a real‐world study of roflumilast demonstrated higher rates of adverse events in patients with COPD than in randomised controlled trials, leading to discontinuation in one‐fifth of patients.^18^

The SLS was a pragmatic trial that evaluated the safety and effectiveness of a novel treatment for COPD, compared with current treatments, in a real‐world setting.^19^ Patient characteristics of the geographically restricted trial population were comparable with the general population of patients with COPD in the UK.^20^ In our present analysis, patients with COPD registered with research‐active practices were representative of all practices contributing to CPRD GOLD and CPRD Aurum (though no direct comparisons between research‐active practices and non‐research‐active practices, and HES‐eligible and non‐HES‐eligible practices were made) in terms of patient characteristics such as age, gender, current smokers, BMI, deprivation and history of respiratory conditions. Study findings using data from research‐active practices could be applied to the overall COPD patient population in both CPRD databases. In our study, a substantial number of patients (203 572) were registered at research‐active practices contributing to CPRD GOLD and CPRD Aurum, providing a large pool of patients with COPD for potential enrolment in real‐world studies. Identification of a large, well‐characterised cohort of patients such as the patients identified in this study, could be used for safety studies, long‐term natural history studies, or comparative effective research, with direct clinical applicability.

We demonstrated that the identification of these “real‐world” study populations is feasible, as the previous extensive work done by van Staa et al,^21^ showed when evaluated the feasibility of point‐of‐care trials with two pilot trials. However, some significant barriers still exist especially regarding privacy concerns, where there is a need for further guidance in the relationship between data protection and scientific research.^22^ Under the 2019 NHS resolution,^23^ primary care contracts for general practitioners (GPs) have now changed and underscore the importance of supporting research. The use of real world data is essential for sound coverage and reimbursment decisions. Although some improvement has been made, a wider appreciation that clinical research is essential to inform patient‐centred clinical practice persists, and more future clinical trials based on real‐world data are needed.

### 4.1 Strengths and limitations

This study demonstrated several strengths of using CPRD data, including a comparatively large sample size, generalizability to the UK population, and detailed information on most patient characteristics relevant to a COPD trial. Identification of a large, well‐characterised cohort of patients with COPD from the CPRD databases at locations across the UK reduces the recruitment burden for real‐world trials and increases the recruitment pool. A further advantage of using CPRD data is that it can be linked to secondary data (HES) to provide a fuller picture of the patient‐care record. Both, CPRD GOLD and CPRD Aurum can be linked to HES, with approximately 56% of practices in CPRD GOLD and nearly all practices in CPRD Aurum (all based in England at the time of this study) are eligible for linkage to HES. However, limitations common to routinely collected primary‐care data persist, including missing data, the risk of misclassification, and a lack of data on whether prescribed medications were dispensed or used.

Direct comparisons between research‐active practices and non‐research active practices, and HES eligible and non‐HES eligible practices were outside the scope of this study. While CPRD GOLD included practices from England, Wales, Northern Ireland, and Scotland, at the time of analysis, CPRD Aurum only included practices from England; practices from Northern Ireland have since begun contributing to CPRD Aurum (since February 2019). The latest set of CPRD linkage data released in spring 2019, after the time of analysis, included an update to linked HES coverage (up to November 2018) for 800 CPRD Aurum practices (increased from 232 in the previous data set).

Additional investigations on the differences in source populations, recording practices and analytical methods will provide further evidence on comparability of CPRD GOLD and CPRD Aurum. However, the results from this study suggest that any unexplored differences are unlikely to affect the validity of potential future clinical trials based on these data.

### 4.2 Conclusion

In conclusion, data from CPRD GOLD and CPRD Aurum were shown to be comparable across key aspects relevant to a COPD trial. Using both CPRD GOLD and CPRD Aurum databases to recruit patients with COPD from a real‐world setting is scientifically feasible. The large, well‐characterised cohort of patients with COPD identified in this study could be used for safety studies, long‐term natural history studies, or comparative effective research, reducing the recruitment burden for real‐world trials and increasing the recruitment pool, and providing data with direct clinical applicability.

## CONFLICT OF INTEREST

Achim Wolf, Daniel Dedman, Rachael Williams and Tarita Murray‐Thomas are full time employees of CPRD, which received funding from GlaxoSmithKline plc. for access to data and services for this study, and receives similar funding from other organisations, including Imperial College. At the time of study conduct, Gema Requena and Jeanne M. Pimenta were full time employees of GlaxoSmithKline plc. and own stocks in the company; Jeanne M. Pimenta has since left GSK. Jennifer K. Quint has nothing to disclose.

## ETHICS STATEMENT

The study used the CPRD database of pseudonymized patient electronic healthcare records therefore patients' informed consent was not required. The study protocol was approved by GSKs Protocol Review Committee and by CPRD Independent Scientific Advisory Committee (ISAC) ISAC protocol number 17_066A.

## AUTHOR CONTRIBUTIONS

Daniel Dedman, Jeanne M. Pimenta and Jennifer K. Quint were involved in study conception/design; Achim Wolf, Rachael Williams, Daniel Dedman and Tarita Murray‐Thomas were involved in data acquisition; all authors were involved in data analysis and/or interpretation. All authors were involved in writing/critical review of draft versions of this manuscript and all approved the final version for submission for publication.

## Supporting information


**Data S1.** Supporting information.
**Table S1.** Description of study variables, associated data sources and method of assessment.
**Table S2.** Reasons for loss to follow up in a sub‐cohort of COPD patients who were actively registered in CPRD practices on 31 December 2012 and number and of patients who remained actively registered at their GP practice after 31 December 2012.
