# Supplementary material for: Feasibility of using Clinical Practice Research Datalink data to identify patients with chronic obstructive pulmonary disease to enrol into real‐world trials
Source: Pharmacoepidemiol Drug Saf. 2021 Jan 9;30(4):472–81. doi: 10.1002/pds.5188 (PMC7986187; doi:10.1002/pds.5188)
Supplement: Supplementary file 1 — Data S1. Supporting information. Table S1. Description of study variables, associated data sources and method of assessment. Table S2. Reasons for loss to follow up in a sub‐cohort of COPD patients who were actively registered in CPRD practices on 31 December 2012 and number and of patients who remained actively registered at their GP practice after 31 December 2012. [file PDS-30-472-s001.docx]

**Supplementary Material**

**SUPPLEMENTARY TABLE 1** Description of study variables, associated data sources and method of assessment.

| **Variables**  **category** | **Description** |
| --- | --- |
| Practice characteristics | The total number of practices, practices with research-active status at time of analysis and practices eligible for HES linkage were reported for all practices with at least one patient. |
| Patient demographics | Variables were age, gender and smoking status on enrolment date, BMI on or ≤90 days before enrolment date, and small area level IMD. IMD quintiles were used for England (2015), Northern Ireland (2010), Scotland (2012) and Wales (2014). IMD data were linked to CPRD primary care data through practice postcodes; linked via lower layer Super Output Area in England, Super Output Area in Northern Ireland and datazone in Scotland.^1^ IMD could not be compared between countries as it measures relative deprivation within each individual country. |
| Respiratory history | Covariates were asthma, bronchiectasis and pneumonia diagnoses, measured as binary variables (yes/no) in all available patient history on or prior to enrolment date. |
| Disease severity | Covariates were dyspnoea (data only reported in patients with exercise limitation; MRC dyspnoea score grades 3–5) recorded on enrolment date or closest record to enrolment date within the previous year, and lung function/airflow limitation (data only reported in patients with severe or very severe disease; GOLD grades 3–4, defined using the GOLD 2006 definitions^2^) and FEV_1_/FVC ratio recorded on enrolment date or the closest record to the enrolment date within the previous 2 years. Acute exacerbations of COPD were identified from the primary care record, using a validated algorithm^3^ based on prescriptions for COPD-specific treatments with antibiotics combined with oral corticosteroid and/or medical diagnosis codes for COPD exacerbations, acute bronchitis, or COPD requiring hospitalisation. Number of exacerbation episodes recorded in year on or prior to the enrolment date were reported. For the subset of linked patients, COPD-related admissions were identified in the linked HES data. |
| Respiratory medication | Variables were initiation of LAMA, LAMA/LABA, ICS/LABA and ICS or ICS/SABA in the 6 months prior to or on the enrolment date. |
| Health resource utilisation | Outcomes were assessed by the number of: GP visits, practice nurse visits and patients having ≥1 recorded flu vaccination in the year prior to or on the enrolment date. The number of all-cause hospitalizations recorded in primary care up to 365 days prior to the trial enrolment date were defined in CPRD GOLD as a Read code for hospitalizations documented in the patient clinical or referral files OR a referral event where the referral type was "In Patient" or "Day Case” OR consultation events where the consultation type was "Discharge Details", “Hospital Admission” or "Casualty Attendance". In CPRD Aurum this was implemented as a Read code for hospitalizations documented in the Observation table OR consultation events where the consultation type was categorised as “Inpatient”, "Discharge Details", “Hospital Admission”, "Casualty Attendance" and “Day Case”. The number of hospital admissions, including days spent in critical care in the year prior to enrolment as recorded in the HES admitted patient care data, was assessed in both CPRD GOLD and CPRD Aurum sub-cohorts eligible for linkage to HES. |
| Other comorbidities at baseline | Covariates were myocardial infarction, stroke and coronary artery bypass grafts, defined by a clinical code in all available history prior to or on the enrolment date. |

BMI, body mass index; COPD, chronic obstructive pulmonary disease; IMD, Index of Multiple Deprivation; GOLD, Global Initiative for Chronic Obstructive Lung Disease; MRC, Medical Research Council; SABA, short-acting beta-2 agonist

CPRD, Clinical Practice Research Datalink; FEV_1_, forced expiratory volume in one second, FVC, forced vital capacity; GP, general practitioner; HES, hospital episode statistics; ICS, inhaled corticosteroid; LABA, long-acting beta-2 agonist; LAMA, long-acting muscarinic antagonist.

**SUPPLEMENTARY TABLE 2** Reasons for loss to follow up in a sub-cohort of COPD patients who were actively registered in CPRD practices on December 31, 2012 and number and of patients who remained actively registered at their GP practice after December 31, 2012.

|  | **CPRD GOLD** | **CPRD Aurum** | **Total** |
| --- | --- | --- | --- |
| **End of study** | 28,711 (63) | 158,710 (68) | 187,421 (67) |
| **Death** | 12,377 (27) | 43,980 (19) | 56,357 (20) |
| **Transfer out** | 4,852 (11) | 32,414 (14) | 37,266 (13) |

Data reported are n (%).

COPD, chronic obstructive pulmonary disease; CPRD, Clinical Practice Research Datalink; GP, general practitioner.

**References**

1. CPRD. CPRD linked data. Accessed 15 June, 2020. <https://www.cprd.com/linked-data>

2. GOLD. Global strategy for the diagnosis, management, and prevention of chronic obstructive pulmonary disease. 2006. Accessed 19 February 2020. <https://www.who.int/respiratory/copd/GOLD_WR_06.pdf>

3. Rothnie KJ, Mullerova H, Hurst JR, et al. Validation of the Recording of Acute Exacerbations of COPD in UK Primary Care Electronic Healthcare Records. *PloS One*. 2016;11(3):e0151357. doi:10.1371/journal.pone.0151357
